# Physical Activity-Based Methodologies as a Physical Education Resource for Inhibitory Control: A Systematic Review with Meta-Analysis

**DOI:** 10.3390/jintelligence14070130

**Published:** 2026-07-01

**Authors:** Eduardo Melguizo-Ibáñez, Pilar Puertas-Molero, Gabriel González-Valero, José Manuel Alonso-Vargas

**Affiliations:** 1 Department of Specific Didactics, Faculty of Education, University of La Laguna, 38200 San Cristóbal de La Laguna, Spain; emelguiz@ull.edu.es (E.M.-I.); alonsojm@ugr.es (J.M.A.-V.); 2 Department of Didactics Musical, Plastic and Corporal Expression, Faculty of Education Science, University of Granada, 18071 Granada, Spain; pilarpuertas@ugr.es

**Keywords:** primary education, early childhood education, executive function, physical activity class, active breaks

## Abstract

Physical activity-based methodologies have been proposed as educational tools to improve executive functions, although their impact on early years and primary education has not yet been fully assessed. This study aims (a) to conduct a systematic review of intervention programmes applying physically active methodologies to inhibitory control in early childhood and primary education and (b) to calculate the overall effect size of intervention programmes applying physically active methodologies to inhibitory control. A systematic search was conducted in the Scopus, Web of Science, Eric and PsycINFO databases. The search yielded a total of 16 scientific articles that formed the basis of the quantitative synthesis. The meta-analysis showed a small overall effect size (g = 0.25; 95% CI [0.11; 0.39] *p* = 0.0006). Meta-regression analyses indicated that the type of intervention and session length were not significantly associated with the effect size. It was found that the number of sessions was significantly associated with the effect size (β = 0.0017; *p* = 0.010). As a conclusion, physically active methodologies may be associated with small improvement in inhibitory control; the certainty of evidence is very low. Confidence in these findings is limited, and the true effect may differ.

## 1. Introduction

Educational institutions play a fundamental role in the holistic development of students, as they provide a space where key skills and abilities are built and developed (Saracho, 2023). Educational institutions not only impart knowledge, but also transform it into practical skills applicable to various situations related to the teaching–learning process (Darling-Hammond et al., 2024). One of the most worrying issues associated with the school environment is the high level of sedentary behaviour, as it has been shown that over 90% of school time is spent on sedentary activities (Cervera-Ramírez et al., 2026; Contardo-Ayala et al., 2024; Fernández-Ortega & Fernández-Revelles, 2026). The scientific literature highlights the need to rethink school dynamics and introduce methodologies that combine physical movement with the teaching–learning process (Contardo-Ayala et al., 2024; Egan et al., 2019; Luna-Pérez, 2026).

Physically active methodologies are defined as innovative teaching tools that incorporate physical movement into the teaching–learning process (González-Pérez et al., 2025). This type of methodology helps students to simultaneously integrate the teaching–learning process with their emotional, physical and cognitive development (González-Pérez et al., 2025; Vazou & Mavilidi, 2021). When applying these methodologies in the classroom, physical activity and academic tasks can take place simultaneously, in series, or sequentially (Layne et al., 2021; van den Berg et al., 2019). These types of methodologies are grounded in the theoretical framework of embodied cognition (Macrine & Fugate, 2022). This framework posits that the human body and the environment are linked to cognitive processes, with the mind being present in the body’s various sensorimotor systems (Macrine & Fugate, 2022). Cognition is fundamentally grounded in sensorimotor experience. The perception and action are not peripheral to thinking but constitute the very mechanism in which cognitive representations are formed (Melguizo-Ibáñez et al., 2026).

Embodied cognition challenges traditional views of cognition as a disembodied process, instead proposing that cognitive activity is distributed across bodily states, environmental contexts and action systems (Zou et al., 2025). Motor activity is not merely an add-on to learning, but an integral component of cognitive processing (Zhang et al., 2025). This theoretical position has important implications for pedagogy, particularly in physical education and classroom-based movement interventions. Learning processes can be enhanced when academic content is intentionally coupled with bodily movement, as this alignment promotes deeper encoding of information through multisensory and motor-based pathways (Melguizo-Ibáñez et al., 2026; Zhang et al., 2025; Zou et al., 2025).

Among physically active methodologies, physically active lessons and active breaks are particularly noteworthy. Physically active lessons are characterised by the deliberate integration of movement into curricular activities, such that academic content is taught through motor-based activities (Barboza et al., 2021). Active breaks, on the other hand, are brief interruptions during the teaching–learning process in which light or moderate physical activity is carried out (Melguizo-Ibáñez et al., 2024). Despite this definition, some research incorporates the teaching of content whilst the active break is taking place (F. Egger et al., 2018; Layne et al., 2021). Both strategies have gained prominence in the design of healthier, more dynamic and inclusive school environments and form part of an educational approach centred on the holistic well-being of students (Booth et al., 2020). In addition to the benefits mentioned above, there is growing evidence linking physically active approaches to improved executive function (Mavilidi et al., 2020; Muñoz-Parreño et al., 2021).

The regulation of various executive functions plays a fundamental role in ensuring meaningful learning (Layne et al., 20201. Executive functions are defined as a series of interrelated cognitive processes that play a key role in the organisation and regulation of learning (Melguizo-Ibáñez et al., 2024). Executive functions are subdivided into three fundamental dimensions (Graham et al., 2021). The first dimension refers to the ability to update and retain relevant information in working memory (Schmidt et al., 2020). The second dimension refers to the ability to switch between multiple operations, tasks, rules and perspectives (Schmidt et al., 2020). The third dimension is termed inhibition and refers to the avoidance of impulsive responses (Schmidt et al., 2020).

Inhibitory control is essential during the teaching–learning process, as it enables students to suppress impulses or distractions in favour of behaviours geared towards academic goals (Schmidt et al., 2020; Jäger et al., 2015). This ability is key to maintaining sustained attention, self-regulating behaviour and managing emotions that could interfere with learning, such as frustration or impulsivity (Konijnenberg & Fredriksen, 2018). In early years and primary education, the development of inhibitory control is particularly important due to the immaturity of the executive system (Bai et al., 2022). In this context, physically active methodologies are presented as effective pedagogical tools for stimulating this executive function (Mavilidi et al., 2020; Muñoz-Parreño et al., 2021). Furthermore, the literature indicates that the combination of movement and cognition at these ages not only contributes to the improvement of executive functions but also enhances the acquisition of meaningful learning (Konijnenberg & Fredriksen, 2018; Mavilidi et al., 2020; Muñoz-Parreño et al., 2021).

Early childhood and primary education represent critical developmental stages for inhibitory control due to the high neurocognitive plasticity observed during these periods (Ferguson et al., 2021; Theodoraki et al., 2020). Previous evidence has shown that inhibitory control develops rapidly between the ages of 4 and 14 (Theodoraki et al., 2020), and it is associated with academic achievement, behavioural self-regulation and socio-emotional adjustment (Davidson et al., 2006). Consequently, interventions implemented during these educational stages may generate greater long-term cognitive and academic benefits than interventions applied later in development (Melguizo-Ibáñez et al., 2024). School settings during early childhood and primary education are characterised by prolonged sedentary time, making them especially suitable contexts for implementing physically active methodologies (Melguizo-Ibáñez et al., 2024; Luna-Villouta et al., 2025).

Although early childhood and primary education differ in curricular structure, both educational stages share important characteristics that justify their joint examination. First, inhibitory control undergoes substantial development through the ages of 4 and 14, making both stages particularly sensitive periods for interventions targeting executive functions (Davidson et al., 2006; Theodoraki et al., 2020). Despite differences in educational aims, both settings are characterised by high levels of sedentary behaviour and an increasing interest in movement-based pedagogies integrated into classroom learning (Melguizo-Ibáñez et al., 2026). Furthermore, physically active methodologies such as active breaks and physically active lessons are implemented in both educational stages with the common purpose of combining movement with cognitive engagement during teaching–learning process (Zurita-Ortega et al., 2025).

Previous systematic reviews and meta-analyses have examined the relationship between physical activity interventions and executive functions in educational settings (de Greeff et al., 2016; Zurita-Ortega et al., 2025). For example, Melguizo-Ibáñez et al. (2024) analysed the effects of active breaks on executive functions in school contexts. Similarly, Zurita-Ortega et al. (2025) evaluated educational proposals based on cognitively demanding exercise and active breaks. This study highlights the potential of movement-based strategies for improving executive functions (Zurita-Ortega et al., 2025). However, these reviews primarily focused on executive functions as a global and did not specifically analyse inhibitory control as an independent cognitive dimension.

Moreover, previous reviews have generally combined heterogeneous interventions such as physical education programmes, extracurricular physical activity, exergames and classroom-based movement interventions. To date, no systematic review with meta-analyses has specifically examined the effects of physically active lessons and active breaks on inhibitory control in early childhood and primary education. Therefore, the present study aims to address this gap by synthesising the available evidence and quantifying the overall effect of physical active methodologies on inhibitory control.

This research has the following objectives:

(a) To conduct a systematic review of intervention programmes that apply physically active methodologies to inhibitory control in pre-school and primary education.

(b) To calculate the overall effect size of intervention programmes using physically active methodologies on inhibitory control in pre-school and primary education.

(c) To examine whether study and intervention characteristics significantly influence the observed effect sizes of physically active methodologies on inhibitory control.

## 2. Materials and Methods

### 2.1. Research Methodology

The systematic review was conducted in accordance with the guidelines set out in the PRISMA statement (Page et al., 2021) (Supplementary Table S1). The review protocol was not registered in any publicly accessible database. This should be considered a limitation of the present study. However, the study followed the PRISMA 2020 guidelines to ensure transparency and methodological rigour throughout the review process.

### 2.2. Eligibility Criteria

The PICOT criteria (Riva et al., 2012) were used to determine the inclusion criteria. Table 1 sets out the inclusion and exclusion criteria in accordance with the PICOT framework.

### 2.3. Literature Review

**Figure 1:**
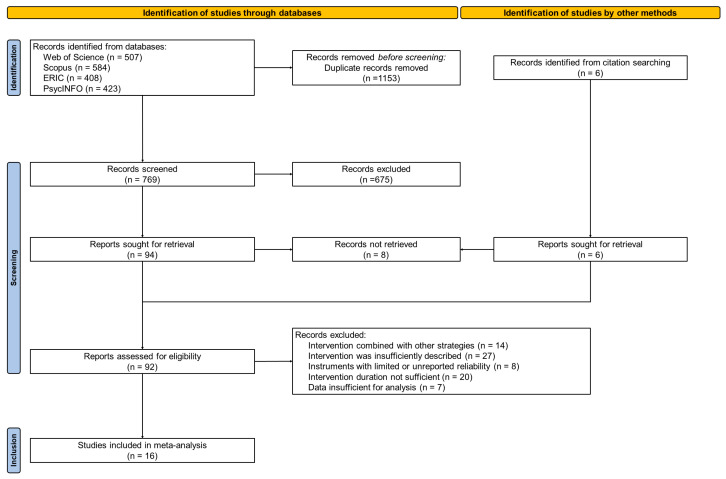
Flowchart of the study.

The literature search was conducted between December 2025 and March 2026. The search was carried out in Web of Science, Scopus, ERIC and PsycINFO. The search strategy was supplemented by manual searches of the existing literature. Table 2 presents the search terms used for each of the databases:

Figure 1 shows the flowchart for the systematic review. The search for scientific studies was conducted by identifying studies through databases and other methods. During the identification phase, a total of 1922 records were retrieved from four databases: Web of Science (n = 507), Scopus (n = 584), Eric (n = 408) and APA PsycINFO (n = 423). After removing 1153 duplicate records, 769 studies proceeded to the screening process.

Two reviewers independently assessed the titles and abstracts to determine eligibility according to predefined criteria. Discrepancies were resolved through discussion, and a third reviewer was consulted when consensus could not be reached. Selection decisions were based on predefined eligibility criteria, which included language, document type, study design, participant characteristics, educational context, type of intervention and outcomes of interest.

During the screening phase, 675 records were excluded for the following reasons: not being written in Spanish, English or Portuguese (n = 39), not being peer-reviewed research (n = 485), not having a randomised design with pre- and post-test data (n = 22), not being conducted on students aged between 4 and 14 (n = 23), conducted in populations with dementia, ADHD or cognitive/genetic impairment (n = 7), not conducted in the classroom (n = 57), not applying an intervention based on active breaks or physically active lessons (n = 29) and not presenting variables of interest (n = 13). As a result, 94 articles were considered potentially relevant and attempts were made to retrieve their full text. Furthermore, through a manual search of the references, 6 additional studies were identified and evaluated in full text. Of these 100 articles, 8 studies could not be retrieved.

During the eligibility phase, a total of 92 full-text articles were assessed. Of these, 76 were excluded for the following reasons: intervention combined with other strategies (n = 14), insufficient description of the intervention (n = 27), instruments with limited or unreported reliability (n = 8), insufficient duration (n = 20) and insufficient data for analysis (n = 7). Finally, 16 studies met the inclusion criteria and were included in the quantitative synthesis.

### 2.4. Assessment of Risk of Bias

Two researchers assessed the risk of bias in each study. In the event of disagreement, a third researcher was brought in to carry out a new assessment and engage in a discussion process to reach a consensus. Due to the review including both RCTs and CTs, design-specific instruments were used to ensure an appropriate appraisal of internal validity. RCTs were evaluated using the Cochrane Risk of Bias 2 (RoB 2) (Sterne et al., 2019). It evaluates five domains: bias arising from the randomization process, deviations from intended interventions, missing outcome data, measurement of outcomes and selection of the reported results (Sterne et al., 2019). Each domain was rated as “low risk”, “some concerns” or “high risk” (Sterne et al., 2019). Non-randomised studies were evaluated using the ROBINS-I tool (Sterne et al., 2016). It assesses seven domains: confounding, participant selection, classification of intervention, deviations from intended intervention, missing data, outcome measurement and selective reporting (Sterne et al., 2016). Judgements were classified as “low”, “moderate”, “serious” or “critical” risk of bias (Sterne et al., 2016).

Publication bias and the effects of small-scale studies were assessed by visually inspecting funnel plots. Funnel plots and Egger’s regression test (M. Egger et al., 1997) were used to examine the possible presence of publication bias.

The influence of individual studies was assessed by calculating the Cook’s distance (Cook, 1977). An F-value of 0.5 (p, n-p) was used as the cut-off point, where p represents the number of model parameters and n the total number of studies (Harrer et al., 2021). Studies whose Cook’s distance exceeded the threshold were considered influential and were excluded from further analysis to examine their impact on the estimation of the overall effect. In addition, standardised residuals were analysed to identify potential outliers. Studies with absolute standardised residuals greater than 2 were considered potentially outliers (Viechtbauer & Cheung, 2010).

### 2.5. Assessment of Heterogeneity

To assess the degree of heterogeneity, the *Q* statistic was used together with the corresponding p-values (Borenstein et al., 2021). It has been reported that the *Q* statistic is quite conservative (Borenstein et al., 2021). For this reason, the level of statistical significance has been set at *p* < 0.1 (Borenstein et al., 2021). Furthermore, it becomes less accurate when the number of available studies is low (Borenstein et al., 2021).

To assess the degree of heterogeneity more comprehensively, the *I*^2^ statistic was also used (Borenstein et al., 2021). The value of this statistic allows the degree of heterogeneity to be classified on a scale from 0 to 100 (Borenstein et al., 2021). The classification thresholds for heterogeneity were set at 25%, 50% and 75% to indicate a low, moderate or high level of heterogeneity, respectively (Borenstein et al., 2021). Discrepancies were resolved through discussion and a third reviewer was consulted if consensus could not be reached. Inter-rater reliability was assessed using Cohen’s kappa, yielding a value of 0.84, indicating a strong agreement between reviewers.

### 2.6. Data Analysis and Calculation of the Effect Size

A random-effects meta-analysis was conducted to combine the effect sizes from each of the studies. The choice of the random-effects model is justified by the degree of heterogeneity among the different studies (Borenstein et al., 2021). As each study differs in aspects such as the characteristics of the intervention and the population, it is assumed that the studies do not share a single true effect, but rather that each estimates a different effect within the distribution of effects (Borenstein et al., 2021).

Effect sizes were calculated based on the difference in standardised means between the experimental and control groups following the intervention, expressed in terms of standard deviations (Cuijpers et al., 2017). The data were extracted from the original studies. Most of the extracted data comprised mean values, standard deviations and the number of participants in both the control and experimental groups. The Comprehensive Meta-Analysis (CMA, version 3; ©2014, Biostat, Inc., Englewood, NJ, USA) software was used. These were calculated using the following data: mean values, number of participants and standard deviation. Effect size was classified into four levels (Cohen, 1998): null (≤0.20), small (0.21–0.49), moderate (0.50–0.79) and large (≥0.80).

Meta-regression analyses of random effects were conducted to examine whether specific intervention characteristics moderated the pooled effect sizes. The models were estimated using restricted maximum likelihood. Regression coefficients (β), standard error (SE), 95% confidence intervals (CI), Z values, and two-sided *p* values were reported for each covariate. The covariates were specified a priori and examined in separate single-moderator models. For categorical moderator, the coefficient represented the mean difference in effect size relative to the reference category. For continuous moderators, the coefficient represented the change in Hedges’g per-unit increase in the moderator.

### 2.7. Analysis of the Certainty of the Evidence

The certainty of the evidence for each outcome was assessed using the GRADE Working Group framework (Schünemann et al., 2008). This framework assesses the overall quality of the evidence across five domains: risk of bias, inconsistency, indirectness, imprecision and publication bias. Evidence derived from RCTs was initially considered to be of high certainty and was subsequently downgraded when relevant methodological limitations were identified. Risk of bias was assessed based on potential methodological issues identified in the included studies, relating to blinding procedures, allocation concealment and intervention fidelity. Inconsistency was assessed by examining the variability in effect sizes across studies. This was examined by interpreting heterogeneity statistics, such as the I^2^ statistic. Indirectness was assessed by considering the applicability of the population, interventions and outcomes to the research questions. Imprecision was assessed based on the sample size of the included studies and the presentation and width of the confidence intervals. Finally, publication bias was considered by assessing the possibility that studies reporting positive effects were more likely to be published. Based on these criteria, the certainty of the evidence for each outcome was classified as high, moderate, low or very low in accordance with the GRADE recommendations (Schünemann et al., 2008).

## 3. Results

### 3.1. Analysis of the Level of Bias

The risk of bias assessment revealed important methodological limitations across both non-randomised (Table 3) and randomised studies (Table 4).

Regarding non-randomised studies (Table 3), most were classified as presenting moderate to serious risk of bias. The primary source of bias was related to confounding, mainly due to the inherent complexity of educational settings. Factors such as classroom environment and student motivation are difficult to control. Additionally, selection bias was present in several studies due to unclear or non-random allocation procedures. Although interventions were generally well-defined, deviations from intended interventions were frequently identified, as implementation often depends on teachers and varies across contexts. In contrast, missing outcome data was consistently rated as low risk, indicating adequate reporting and handling of attrition. However, measurement bias remained a concern due to the absence of blinding in outcome assessment. Selective reporting bias was frequently rated as moderate, as most studies did not report pre-registration protocols.

For randomised studies (Table 4), all studies were classified as having an overall high risk of bias, although this result should be interpreted cautiously. The main limitation was related to bias due to deviations from intended interventions. Blinding of participants and personnel was not feasible in school-based interventions where the intervention is inherently visible. This limitation is structural rather than methodological and it affects research conducted in real-world educational contexts. Additionally, most studies presented some concerns in the randomization process, primarily due to the lack of reporting on allocation concealment. Measurement bias was also frequently identified due to the absence of blinded outcome assessor. In contrast, missing data bias was generally low, as most studies reported minimal attrition. Selective reporting bias was typically rated as “some concerns” due to the lack of protocol preregistration in many studies.

**Figure 2:**
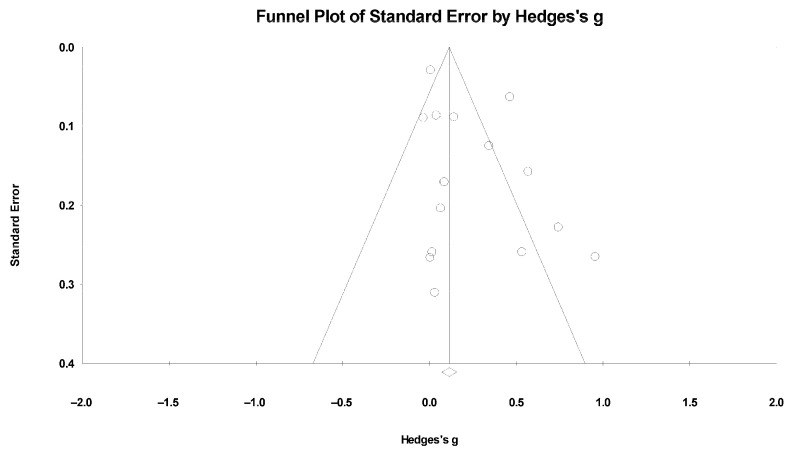
Funnel plot.

The funnel plot (Figure 2) shows the distribution of effect sizes as a function of their standard errors. Most studies are concentrated in the upper part of the plot, with relatively low standard errors and small positive effect sizes. The graph shows a slight right-skewed distribution, evidenced by the presence of several studies with moderate and large effect sizes. This skewness is reflected in the uneven distribution of studies around the line representing the average effect size, with a greater concentration on the positive side and a scarcity of studies with negative or null effects.

Egger’s regression test was conducted to statistically assess funnel plot asymmetry. The intercept was statistically significant (*intercept* = 1.87, *SE* = 0.74, *t* = 2.53, *p* = 0.023), suggesting the presence of small-study effects. Although these findings may indicate potential publication bias associated with the absence of small studies reporting non-significant or negative results, they should be interpreted with caution. Given the relatively small number of included studies (n = 16) and the high level of heterogeneity (χ^2^ = 82.11; *df* = 15, *p* < 0.00001; *I^2^* = 82%), the observed asymmetry may reflect true between-study heterogeneity or small-study effects rather than publication bias alone.

### 3.2. Level of Certainty

The assessment of the quality of evidence using the GRADE approach (Table 5) revealed that the overall level of evidence is very low. This classification is primarily due to common methodological limitations in the included studies, as well as the high level of heterogeneity observed. The risk of bias was considered serious, as most studies lacked blinding of participants and staff, as well as information on allocation concealment and assessor blinding. These limitations are inherent to physical activity-based educational interventions, where experimental control is more difficult to implement in real-world settings.

A very serious inconsistency was identified, reflected in the high levels of heterogeneity (I^2^ = 82.0%), suggesting considerable variability in the observed effects. This heterogeneity may be explained by differences in the duration, intensity, type of intervention and assessment tool used to evaluate inhibitory control. Conversely, no significant issues were identified in terms of indirect evidence, as the included studies directly addressed the target population and the interventions of interest and used validated instruments. Imprecision was further considered serious due to the lack of adjustment for clustering in several included studies, which may have led to underestimated standard errors and overly narrow confidence intervals. Sample sizes were generally adequate, and estimates were sufficiently precise. Despite this, a possible publication bias was observed, evidenced by the asymmetry of the funnel plot, suggesting a probable under-representation of studies with null or negative results.

### 3.3. Analysis of the Studies

Table 6 presents the following data from the studies: (1) Authors (Year); (2) Design; (3) Educational stage; (4) Participants; (5) Methodology; (6) Instruments; (7) Effect size [95% CI]; (8) Conclusions. Regarding the study design, the majority employ a randomised controlled trial approach (n = 16; 81.25%). It is observed that most studies were conducted in primary education (n = 13; 81.25%). It is also observed that there is greater use of physically active lessons (n = 9; 56.25%) than active breaks (n = 7; 43.75%).

### 3.4. Overall Effect Size

**Figure 3:**
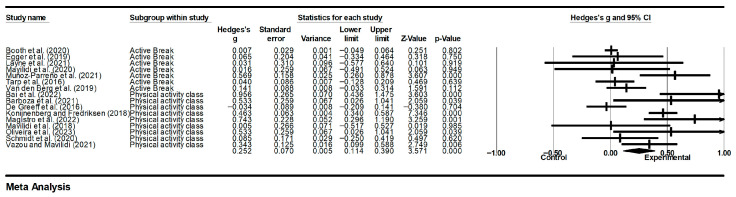
Forest plot diagram of interventions based on physically active methodologies.

Figure 3 shows the effect size of physically active approaches on inhibitory control. A small but significant overall effect is observed (*g* = 0.252; *95% CI* [0.114; 0.390]; *Z* = 3.56; *p* = 0.0006). Heterogeneity statistics indicated substantial variability across studies (*τ*^2^ = 0.05; *Q* = 82.11, *df* = 15, *p* < 0.00001; *I*^2^ = 82%).

### 3.5. Meta-Regression Analysis

Meta-regression analyses were conducted in an exploratory manner to examine whether specific intervention characteristics were associated with variations in effect sizes (Table 7). Compared with active breaks as the reference category, physical active classroom interventions tended to yield larger effects, although this difference did not reach statistical significance (*β* = 0.228; *SE* = 0.125, *95% CI* [−0.017, 0.472], *Z* = 1.83, *p* = 0.068).

The number of sessions was a significant moderator of the effect size (*β* = 0.0017; *SE* = 0.0007, 95% CI [0.0004, 0.0031], *Z* = 2.57, *p* = 0.010), indicating that interventions delivered over a greater number of sessions were associated with slightly larger effects. In contrast, session length was not significantly related to the inhibitory control (*β* = 0.0073; *SE* = 0.0046, *95% CI* [−0.0017, 0.0163], *Z* = 1.59, *p* = 0.111).

## 4. Discussion

The findings of the studies included in the systematic review reveal considerable variability in the effects of physically active interventions on inhibitory control in pre-school and primary school children.

The findings of the studies reveal considerable variability in the effectiveness of implementing active breaks in the classroom. Specifically, it is observed that the frequency and consistency of active break implementation are key factors when applying this type of physically active methodology (Booth et al., 2020; Muñoz-Parreño et al., 2021). Furthermore, proper planning combined with the correct implementation of active breaks can lead to improvements in subjective academic well-being, highlighting a possible combined emotional-cognitive effect of this methodology (Booth et al., 2020; Layne et al., 2021; Tarp et al., 2016). Other studies have failed to produce significant changes, particularly when they were of short duration or when the cognitive demand was excessive (F. Egger et al., 2018; van den Berg et al., 2019). Regarding cognitive load, if this is not appropriate, it can lead to cognitive overload, resulting in difficulties in processing information related to the teaching–learning process (Zurita-Ortega et al., 2025).

It has been shown that physically active lessons have greater benefits for inhibitory control, particularly when academic content is integrated, and the lessons are delivered over a period of time. The application of this type of physically active methodology showed a reduction in errors and response times following a nine-month intervention (Barboza et al., 2021). Benefits were also found when physically active sessions were incorporated into mathematics learning (Magistro et al., 2022). Despite these findings, it has been shown that not all studies found significant improvements, which may be linked to the duration of the intervention or the type of task used in the classroom (de Greeff et al., 2016; Mavilidi et al., 2018).

Similarly, studies that incorporate physically active lessons into early years education do not report significant improvements in inhibition, although they do report improvements in attention (Schmidt et al., 2020; Vazou & Mavilidi, 2021). These results are based on the difficulty of influencing complex executive functions at an early age (Schmidt et al., 2020; Vazou & Mavilidi, 2021). This dissociation may be explained by developmental differences between executive function components. Attentional processes tend to emerge earlier and are more sensitive to environmental stimulation (Vazou & Mavilidi, 2021), whereas inhibitory control is a more complex and later-developing function that requires sustained and targeted practice (Melguizo-Ibáñez et al., 2026). In early childhood, neural systems underlying attention are more malleable and responsive to movement-based and engaging activities (Melguizo-Ibáñez et al., 2026). In contrast, inhibitory control involves higher-order regulation impulses and responses, which may not be sufficiently challenged by short-term or low-intensity interventions (Muñoz-Parreño et al., 2021).

The systematic use of physically active methodologies promotes a restructuring of the roles of teachers and pupils in the classroom (González-Pérez et al., 2025). The teacher acts as a facilitator of learning by designing active teaching experiences that combine curricular content with motor and cognitive challenges, whilst students become active agents of their own learning, which promotes metacognition and intrinsic motivation (Darling-Hammond et al., 2024). The application of these methodologies involves a pedagogical approach centred on well-being, as improvements in inhibitory control are associated with increased emotional and academic well-being (Graham et al., 2021; Muñoz-Parreño et al., 2021).

The results of the meta-analysis show that physically active methodologies have a positive and significant effect on inhibitory control. These findings are supported by previous research that recognises the value of physical movement as a catalyst for improving inhibitory control during childhood (Mavilidi et al., 2020; Muñoz-Parreño et al., 2021). This enables learners to resist distractions, control impulses and focus their attention on specific academic tasks, making it essential in settings where concentration and behavioural regulation are necessary for meaningful learning (Layne et al., 2021; Mazzoli et al., 2021). In this regard, physically active methodologies not only act as motor interventions but also modify the student’s cognitive conditions, facilitating mental processes linked to academic performance (Melguizo-Ibáñez et al., 2024).

No statistically significant differences have been found in the type of active methodology applied to inhibitory control. Unlike active breaks, physically active lessons incorporate movement as part of the curriculum, creating a learning environment in which students learn by doing, moving and experiencing in a multisensory way (Macrine and Fugate, 2022). It has been demonstrated that when physical movement is directly integrated into school content, the benefits extend not only to improved inhibitory control but also to content comprehension, academic motivation and the emotional climate of the classroom (Magistro et al., 2022). This pedagogical integration strengthens the teaching–learning process by fostering dynamic, participatory environments geared towards the student’s holistic development (Bai et al., 2022). Conversely, active breaks, although proven useful for breaking the sedentary routine of the classroom, yielded more inconsistent results. It has been found that active breaks produce significant improvements when they are designed and adapted to the cognitive level of the pupils (F. Egger et al., 2018; Mavilidi et al., 2020).

Although the study’s objectives were met, it should be noted that this research has certain limitations. These include a high degree of heterogeneity among the studies, resulting from differences in design, duration, and intensity of the interventions, as well as the variety of instruments used to measure inhibitory control. Furthermore, many studies showed moderate or uncertain risks of bias, particularly regarding randomisation and the blinding of participants and assessors. It is also important to note that most of the studies were conducted in primary education, with a limited sample in early years education. Although both stages share developmental relevance for executive functions, differences in curricular organisation, teaching approaches and cognitive maturity may partially explain the heterogeneity observed. A further limitation is that variables related to regular physical education provision were not consistently available across the included studies, preventing their control in the analysis. In addition, the review protocol was not prospectively registered in any publicly accessible database, which should be considered a methodological limitation. Despite the low quality of the evidence, the results of the meta-analysis consistently show a positive and significant effect of physically active methodologies on inhibitory control. The findings should be interpreted with caution, as the effect size may differ substantially from the estimate. Finally, the consistency in the direction of the effects supports the relevance of these interventions in the educational setting.

The high level of heterogeneity observed indicates substantial variability across studies, which may be attributed to differences in intervention characteristics. This variability limits the generalisability of the findings. In addition, the asymmetry observed suggests the possible presence of publication bias with an underrepresentation of studies reporting null or negative effects. This bias may lead to an overestimation of the true effect of physically active methodologies on inhibitory control.

The findings obtained are highly applicable in an educational context, as they support the systematic integration of physically active methodologies as a strategy for improving inhibitory control during the teaching–learning process. Their application enables the curriculum to be redesigned by incorporating movement into academic activities, promoting pupils’ emotional and cognitive well-being, reducing sedentary behaviour in schools, and training teachers in innovative practices tailored to both the educational stage and the needs of the group.

## 5. Conclusions

The findings of this systematic review with meta-analysis suggest that physically active methodologies may be associated with small improvements in inhibitory control in early childhood and primary education. However, the certainty of the evidence supporting this effect is very low, which substantially limits confidence in the estimated outcomes.

Although the pooled results indicate a small positive effect, this finding should be interpreted with considerable caution due to important methodological limitations across studies. These factors suggest that the true effect may be substantially different from the observed estimate.

Regarding the type of intervention, physically active lessons showed a tendency toward larger effects compared to active breaks, although this difference was not statistically significant. Therefore, it can not be concluded with confidence that one approach is more effective than another. Overall, physically active methodologies should be considered as promising but not yet well-established educational strategies for improving inhibitory control.

Future research is needed to strengthen the evidence in this field, particularly through well-designed randomised controlled trials. Reducing heterogeneity and addressing potential publication bias will be essential to provide more reliable estimates of effect.

## Figures and Tables

**Table 1:** Inclusion Criteria.

	Inclusion Criteria
**Population**	Pupils in nursery and primary education aged between 4 and 14.
	Healthy students with no cognitive impairments.
**Intervention**	Lessons that incorporate physically active methodologies into the classroom (physically active lessons and active breaks) and that examine inhibitory control.
	Studies conducted in formal educational settings
**Comparison**	Groups that do not use physically active methods or that conduct sedentary classes.
**Outcome**	Measurement of inhibitory control using standardised tests or validated instruments.
**Time**	Studies involving interventions lasting at least three weeks, including assessments at the end of the programme
**Other criteria**	The following statistical data are available for both the control and experimental groups: mean values, standard deviations and the number of participants in both groups.
	Studies with a randomised controlled trial or controlled trial design.
	Availability of pre-test and post-test data.
	Peer-reviewed scientific articles
	Research papers written in Spanish, English and Portuguese

**Table 2:** Search structures in each database.

Database	Search Structure
**Web of Science**	TS = (“physically active” OR “active learning” OR “movement-based” OR “active break*” OR “classroom-based physical activity” OR “physically active classroom*”) AND TS = (“executive function*” OR inhibition OR “inhibitory control” OR “cognitive control” OR attention OR “self-regulation”) AND TS = (child* OR school* OR “primary education” OR “elementary education” OR preschool* OR “early childhood education”)
**Scopus**	TITLE-ABS-KEY (“physically active” OR “active learning” OR “movement-based” OR “active break*” OR “classroom-based physical activity” OR “physically active classroom*”) AND TITLE-ABS-KEY (“executive function*” OR inhibition OR “inhibitory control” OR “cognitive control” OR attention OR “self-regulation”) AND TITLE-ABS-KEY (child* OR school* OR “primary education” OR “elementary education” OR preschool* OR “early childhood education”)
**ERIC and** **PsycINFO (EBSCO)**	(TX(“physically active”) OR TX(“active learning”) OR TX(“movement-based”) OR TX(“active break*”) OR TX(“classroom-based physical activity”) OR TX(“physically active classroom*”)) AND (TX(“executive function*”) OR TX(inhibition) OR TX(“inhibitory control”) OR TX(“cognitive control”) OR TX(attention) OR TX(“self-regulation”)) AND (TX(child*) OR TX(school*) OR TX(“primary education”) OR TX(“elementary education”) OR TX(preschool*) OR TX(“early childhood education”))

**Table 3:** Risk of bias assessment of non-randomised studies.

Study	Confounding	Selection of Participants	Classification of Interventions	Deviations from Intended Interventions	Missing Data	Measurement of Outcomes	Selection of Reported Results	Overall Risk
**Barboza et al.** **(2021)**	Possible teacher and classroom effects not controlled (Moderate)	Unclear random allocation procedure (Moderate)	Intervention clearly defined and described (Low)	Implementation may vary across teachers (Moderate)	Low attrition and adequately reported (Low)	No blinding: Cognitive test may be influenced (Moderate)	No pre-registered protocol reported (Moderate)	Moderate
**Muñoz-Parreño et al.** **(2021)**	Educational context and motivational factor not controlled (Serious)	Allocation process not fully randomised (Moderate)	Active breaks programme well described (Low)	Intervention fidelity dependent on teacher (Moderate)	Low dropout and well managed (Low)	No blinding: Cognitive test may be influenced (Moderate)	No protocol registration reported (Moderate)	Serious
**Oliveira et al.** **(2023)**	Long-term intervention with maturation and contextual confounders (Serious)	Controlled but fully randomised design (Moderate)	Physically active lessons clearly described (Low)	Long duration increases variability in implementation (Moderate)	Attrition adequately controlled (Low)	Outcome assessors likely not blinded (Moderate)	Relatively complete reporting but no clear pre-registration (Moderate)	Serious

**Table 4:** Risk of bias assessment of randomised studies.

Study	Randomization Process	Deviations from Intended Interventions	Missing Outcome Data	Measurement of the Outcome	Selection of the Reported Results	Overall Risk
**Bai et al.** **(2022)**	Allocation concealment not reported despite adequate cluster randomization (Some concerns)	Participants and teachers aware of intervention; no blinding possible (High)	Low attrition and appropriate handling of missing data (Low)	No information on blinding of outcome assessors (Some concerns)	Lack of protocol pre-registration (Some concerns)	High
**Booth et al.** **(2020)**	Randomization procedure not clearly described (Some concerns)	Visible intervention and high variability in implementation fidelity (High)	Insufficient reporting of attrition and missing data (Some concerns)	Non-blinded cognitive assessments (Some concerns)	Lack of protocol pre-registration (Some concerns)	High
**de Greeff et al.** **(2016)**	Cluster randomization described but allocation concealment not reported (Some concerns)	Intervention visible to participants and teachers (High)	Low attrition and well-managed data (Low)	No information on assessor blinding (Some concerns)	Absence of protocol information (Some concerns)	High
**Konijnenberg and Fredriksen** **(2018)**	Randomization described but no allocation concealment information (Some concerns)	Participants aware of intervention (High)	Minimal attrition (Low)	No blinding of assessors (Some concerns)	Incomplete reporting of outcomes (Some concerns)	High
**Layne et al.** **(2021)**	Unclear assignment process (Some concerns)	Open-label design; Contextual influences likely (High)	Incomplete reporting of attrition (Some concerns)	No blinding of assessors (Some concerns)	Incomplete reporting of outcomes (Some concerns)	High
**Magistro et al.** **(2022)**	Unclear assignment process (Some concerns)	Visible intervention; No blinding (High)	Minimal attrition (Low)	Lack of assessor blinding (Some concerns)	No protocol registration (Some concerns)	High
**Mavilidi et al.** **(2018)**	Unclear assignment process (Some concerns)	Visible intervention; No blinding (High)	Minimal attrition (Low)	Open-label outcome measurement (Some concerns)	No protocol registration (Some concerns)	High
**Mavilidi et al.** **(2020)**	Cluster randomization clearly described and appropriate (Low)	Participants aware of intervention (High)	Minimal attrition (Low)	Open-label outcome measurement (Some concerns)	Results reported according to protocol (Low)	High
**Schmidt et al.** **(2020)**	Cluster randomization clearly described and appropriate (Low)	Participants aware of intervention (High)	Minimal attrition (Low)	No blinding of assessors (Some concerns)	Outcomes consistently reported (Low)	High
**Tarp et al.** **(2016)**	Randomization clearly described (Low)	Visible intervention; No blinding (High)	Low levels of missing data (Low)	No blinding of assessors (Some concerns)	Outcomes consistently reported (Low)	High
**van den Berg et al.** **(2019)**	Randomization clearly described (Low)	Participants aware of intervention (High)	Low levels of missing data (Low)	No blinding of assessors (Some concerns)	Secondary outcomes not fully reported (Some concerns)	High
**Mavilidi and Vazou** **(2021)**	Randomization insufficiently detailed (Some concerns)	Visible intervention; No blinding (High)	Minimal attrition (Low)	No blinding of assessors (Some concerns)	Unclear selective reporting (Some concerns)	High

**Table 5:** Analysis of the degree of certainty.

Result	Number of Studies	Design	Risk of Bias	Inconsistency	Circumstantial Evidence	Imprecision	Publication Bias	Effect Size [95% CI]	Overall Quality (GRADE)	Interpretation
**Inhibitory control**	16	RCTs and controlled trials	Serious (no blinding, open-label)	Very serious (I^2^ = 82.0%)	Not serious	Serious	Probable (funnel plot asymmetry)	g = 0.252 [0.114; 0.39]	Very low	A small but highly uncertain positive effect
**Physical activity classes**	9	RCTs and controlled trials	Serious	Very serious (I^2^ = 77.0%)	Not serious	Serious	Probable	g = 0.37 [0.16; 0.58]	Very low	More effective than active breaks, but low confidence
**Active breaks**	7	RCTs and controlled trials	Serious	Very serious (I^2^ = 56.0%))	Not serious	Serious	Probable	g = 0.18 [0.00; 0.36]	Very low	Minor and inconsistent effect

**Table 6:** Findings and conclusions drawn for the research.

Authors (Year)	Design	Duration	Educational Stage	Participants	Methodology	Instruments	Effect Size [95% CI]	Conclusions
**de Greeff et al.** **(2016)**	Randomised controlled trial	22 weeks with 3–4 sessions of 25 min	Primary Education	499 (8.1 ± 0.7)	Physical activity class	Golden Stroop test	−0.03 [−0.21; 0.14]	No significant improvement in inhibitory control was observed.
**Tarp et al.** **(2016)**	Randomised controlled trial	20 weeks, with 5 sessions of 45 min each	Primary Education	632 (12.8 ± 0.6)	Active break	Eriksen Flanker test	0.04 [−0.13; 0.21]	No significant improvement in inhibitory control was observed.
**Konijnenberg and Fredriksen** **(2018**)	Randomised controlled trial	9 months with 5 daily sessions of 15 min	Primary Education	1173 (6–12 years)	Physical activity class	Eriksen Flanker test	0.46 [0.34; 0.59]	Significant improvement in inhibitory control was observed.
**Mavilidi et al.** **(2018)**	Randomised controlled trial	6 weeks, with 2 sessions per week, each lasting 15 min	Primary Education	55 (10.26 ± 0.35)	Physical activity class	Eriksen Flanker test	0.00 [−0.52; 0.53]	No significant improvement in inhibitory control was observed
**F. Egger et al.** **(2019)**	Randomised controlled trial	12 weeks with 3 weekly sessions of 15 min	Primary Education	142 (7.91 ± 0.40)	Active break	Eriksen Flanker test	0.06 [−0.34; 0.47]	No significant improvement in inhibitory control was observed
**van den Berg et al.** **(2019)**	Randomised controlled trial	5 weeks, with 3 sessions of 25 min each week	Primary Education	512 (9–12 years)	Active break	Stroop Task	0.14 [−0.03; 0.31]	No significant improvement in inhibitory control was observed
**Booth et al.** **(2020)**	Randomised controlled trial	6 weeks with a daily 10 min session	Primary Education	5463 (10.2 ± 0.7)	Active break	Signal stop task	0.01 [−0.05; 0.06]	No significant improvement in inhibitory control was observed
**Layne et al.** **(2021)**	Randomised controlled trial	8 weeks with 3 weekly 10 min sessions	Primary Education	40 (8–9 years)	Active break	Go/No-Go test	0.03 [−0.59; 0.65]	No significant improvement in inhibitory control was observed
**Mavilidi et al.** **(2020)**	Randomised controlled trial	6 weeks with 3 weekly sessions of 15 min	Primary Education	87 (9.11 ± 0.62)	Active break	Stroop Task	0.02 [−0.50; 0.53]	No significant improvement in inhibitory control was observed
**Schmidt et al.** **(2020)**	Randomised controlled trial	8 weeks with 3 weekly sessions of 25 min	Early Childhood Education	189 (5.34 ± 0.59)	Physical activity class	Stroop Task	0.08 [−0.25; 0.42]	No significant improvement in inhibitory control was observed
**Barboza et al.** **(2021)**	Controlled trial	9 months with 3 weekly sessions of 50 min	Primary Education	61 (7.8 ± 0.6)	Physical activity class	Go/No-Go test	0.53 [0.02; 1.05]	Significant improvement in inhibitory control was observed
**Muñoz-Parreño et al.** **(2021)**	Controlled trial	12 weeks with 3 weekly 10 min sessions	Primary Education	166 (10.9 ± 0.7)	Active break	NIH-EXAMINER Battery	0.57 [0.26; 0.88]	Significant improvement in inhibitory control was observed
**Mavilidi and Vazou** **(2021)**	Randomised controlled trial	12 weeks with 3 weekly sessions of 15 min	Early Childhood Education	273 (4.22 ± 0.61)	Physical activity class	Go/No-Go test	0.34 [0.10; 0.59]	Significant improvement in inhibitory control was observed
**Bai et al.** **(2022)**	Randomised controlled trial	12 weeks with 3 weekly 40 min sessions	Early Childhood Education	92 (4.44 ± 0.46)	Physical activity class	Silly Sound Stroop Task	0.96 [0.43; 1.48]	Significant improvement in inhibitory control was observed
**Magistro et al.** **(2022)**	Randomised controlled trial	24 months, with two 60 min sessions per week	Primary Education	82 (6.63 ± 0.28)	Physical activity class	Go/No-Go test	0.74 [0.29; 1.19]	Significant improvement in inhibitory control was observed
**Oliveira et al.** **(2023)**	Controlled trial	24 months with two 60 min sessions per week	Primary Education	61 (7.8 ± 0.5)	Physical activity class	Computerised inhibitory response test	0.53 [0.02; 1.05]	Significant improvement in inhibitory control was observed

**Table 7:** Meta-regression analysis.

Predictor	Reference Category	β	SE	95% CI		df	Z	*p*
				Lower	Upper			
Physical Active Classroom	Active Breaks	0.228	0.125	−0.017	0.472	1	1.83	0.068
Duration (Number of Sessions)	-	0.0017	0.0007	0.0004	0.0031	15	2.57	0.010
Duration (Minutes)	-	0.0073	0.0046	−0.0017	0.0163	15	1.59	0.111

## Data Availability

Data sharing is not applicable. No new data were created or analysed in this study.

## Supplementary Materials

The following supporting information can be downloaded at: https://www.mdpi.com/article/10.3390/jintelligence14070130/s1, Table S1. PRISMA 2020 Checklist.
